# Bleeding Downhill Esophageal Varices Associated With Benign Superior Vena Cava Syndrome: A Case Report

**DOI:** 10.7759/cureus.99155

**Published:** 2025-12-13

**Authors:** Linvydas Ivaskevicius, Sena Bluemel, Tim Sebastian, Gabor Forgo, Nils Kucher

**Affiliations:** 1 Department of Angiology, University Hospital of Zürich, Zürich, CHE; 2 Department of Gastroenterology and Hepatology, University Hospital of Zürich, Zürich, CHE

**Keywords:** benign superior vena cava syndrome, downhill esophageal varices, endovascular stent, hemodialysis catheter, upper gastrointestinal bleeding

## Abstract

Downhill esophageal varices are an uncommon source of upper gastrointestinal bleeding and may be easily overlooked when no underlying liver disease is present. In this report, a young patient receiving long-term hemodialysis developed recurrent bleeding episodes caused by severe central venous obstruction secondary to a chronically implanted catheter. Proximal esophageal varices were only identified during a later endoscopic evaluation, which prompted targeted imaging and revealed complete occlusion of the central thoracic veins. The patient subsequently underwent endovascular reconstruction of the obstructed venous pathways, including angioplasty, stenting, and catheter exchange. This intervention resulted in stable venous patency and complete cessation of bleeding during follow-up.

This case highlights the importance of considering superior vena cava-related varices as a potential cause of unexplained upper gastrointestinal bleeding and demonstrates that restoring venous outflow can provide durable clinical resolution.

## Introduction

Esophageal bleeding can be a life-threatening emergency, mostly associated with portal hypertension and liver cirrhosis. Rarely, esophageal bleeding may develop due to superior vena cava (SVC) obstruction, causing “downhill” varices. These varices originate in the upper esophagus and drain caudally, in contrast to the more common “uphill” varices that result from portal hypertension and drain cranially in the esophageal wall.

SVC syndrome is characterized by obstruction of the SVC, resulting in impaired venous return from the upper body to the heart with consequent venous congestion. Obstruction of the SVC raises its pressure, reversing blood flow from the SVC into the esophageal veins and triggering collateral circulation through the esophageal venous plexus. This increased flow dilates the veins, forming varices primarily in the upper third of the esophagus [1]. While malignancies such as lung cancer or lymphoma are the most common causes of SVC syndrome, bleeding from downhill varices is more often linked to benign factors like indwelling central venous catheters or chronic thrombosis [1]. This may be due to anticoagulant use and/or uremic coagulation dysfunctions.

We present a rare case of recurring downhill variceal bleeding secondary to catheter-associated SVC obstruction in a young dialysis patient.

## Case presentation

A 25-year-old female patient with end-stage renal disease (ESRD) secondary to IgA nephropathy was undergoing long-term hemodialysis via a right internal jugular tunneled dialysis catheter that had been in place for more than four years.

Over a period of approximately one and a half years, she experienced three episodes of hospitalization due to upper gastrointestinal bleeding. The initial bleeding episode was associated with a hemoglobin drop from 101 g/L to 64 g/L, with endoscopy showing no active bleeding or identifiable source. A second episode occurred several months later, again presenting with hematemesis and melena but with less pronounced anemia, and repeat endoscopy revealed no evidence of active bleeding or underlying pathology.

**Figure 1:**
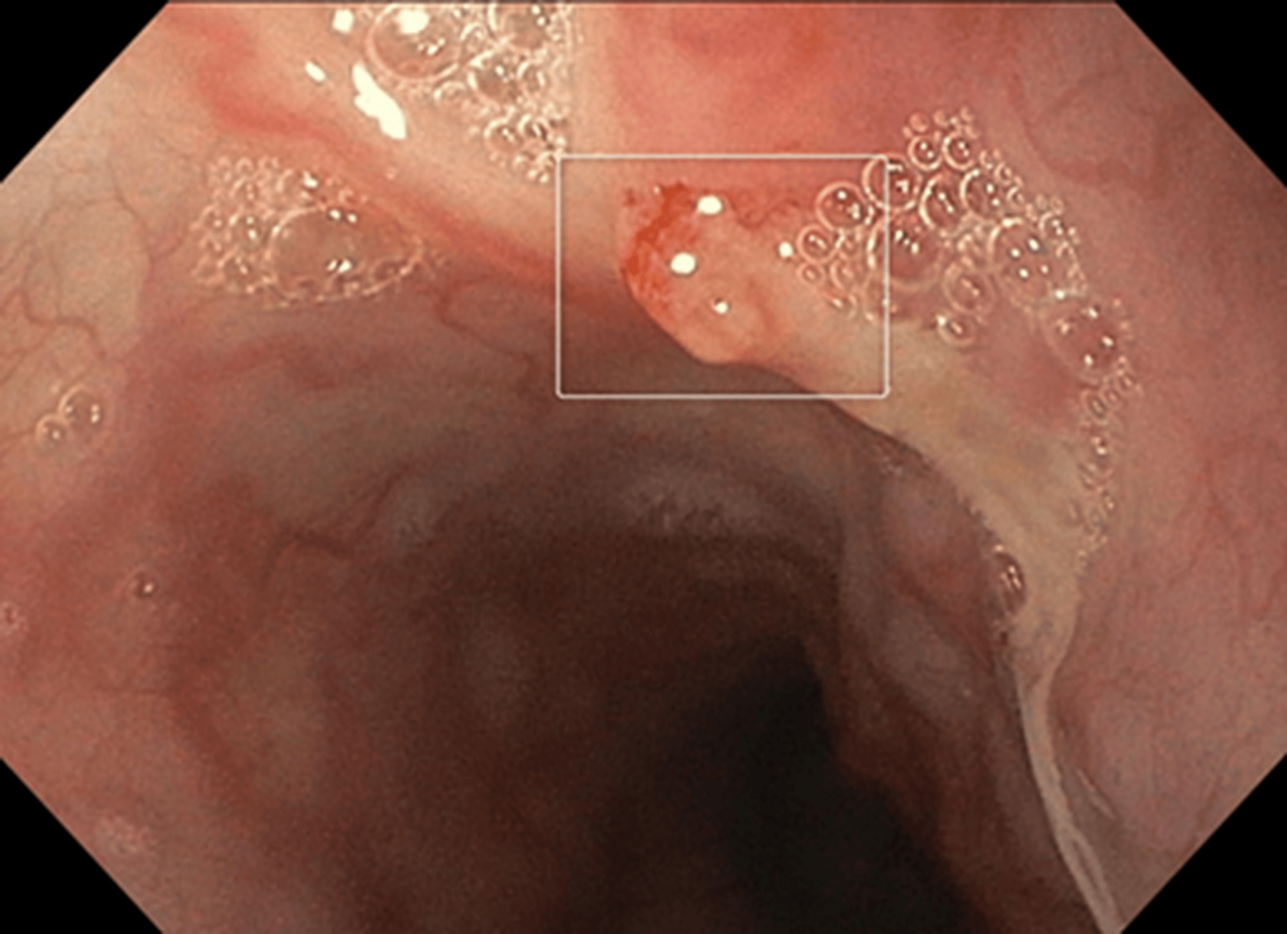
Endoscopic finding showing a "white nipple sign" in the upper esophagus. This represents a protruding white fibrin plug, indicating one potential source of bleeding.

Several months thereafter, she developed a third, more severe episode of upper gastrointestinal bleeding. Endoscopy demonstrated a white nipple sign in the upper esophagus (Figure 1) consistent with a recent variceal hemorrhage, and an endoscopic variceal band ligation was performed. Gastroenterological investigations showed no evidence of portal hypertension and no signs of liver fibrosis or cirrhosis.

**Figure 2:**
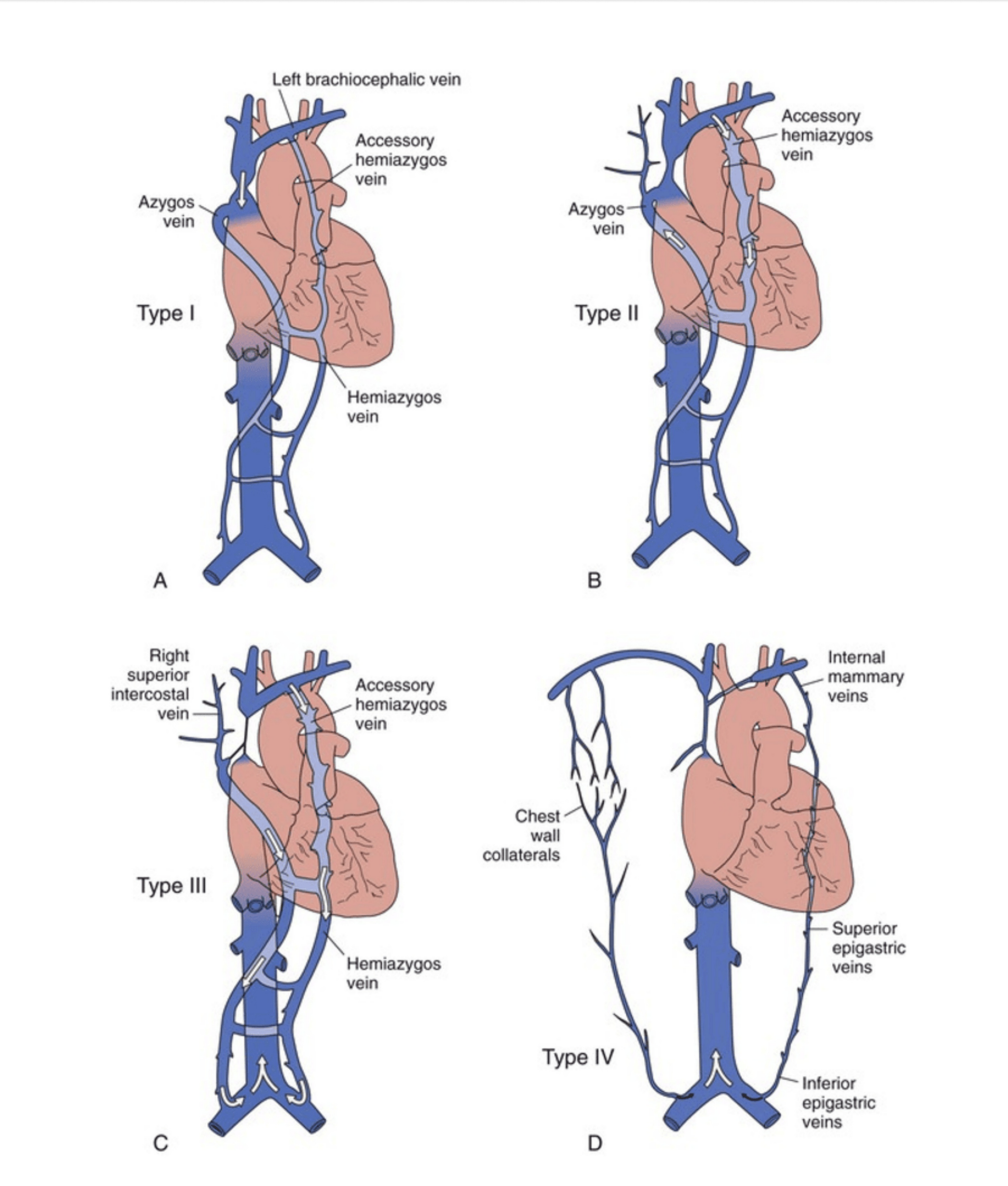
Stanford classification for superior vena cava (SVC) occlusion. Type I - high-grade SVC obstruction (< 90%). Type II - greater than 90% SVC stenosis. Type III - complete occlusion of SVC with retrograde flow in both azygos and hemiazygos veins. Type IV - complete SVC obstruction with prominent collateral flow involving the internal mammary and epigastric veins. Adapted from Reference [2].

Further work-up with contrast-enhanced CT venography showed a complete occlusion of the right internal jugular vein, subclavian vein, both brachiocephalic veins, and the SVC with an indwelling dialysis catheter. A corresponding schematic illustration of SVC syndrome aligned with the Stanford classification [2] is included to facilitate a clearer understanding of the underlying venous anatomy and the extent of the occlusive pathology (Figure 2). 

**Figure 3:**
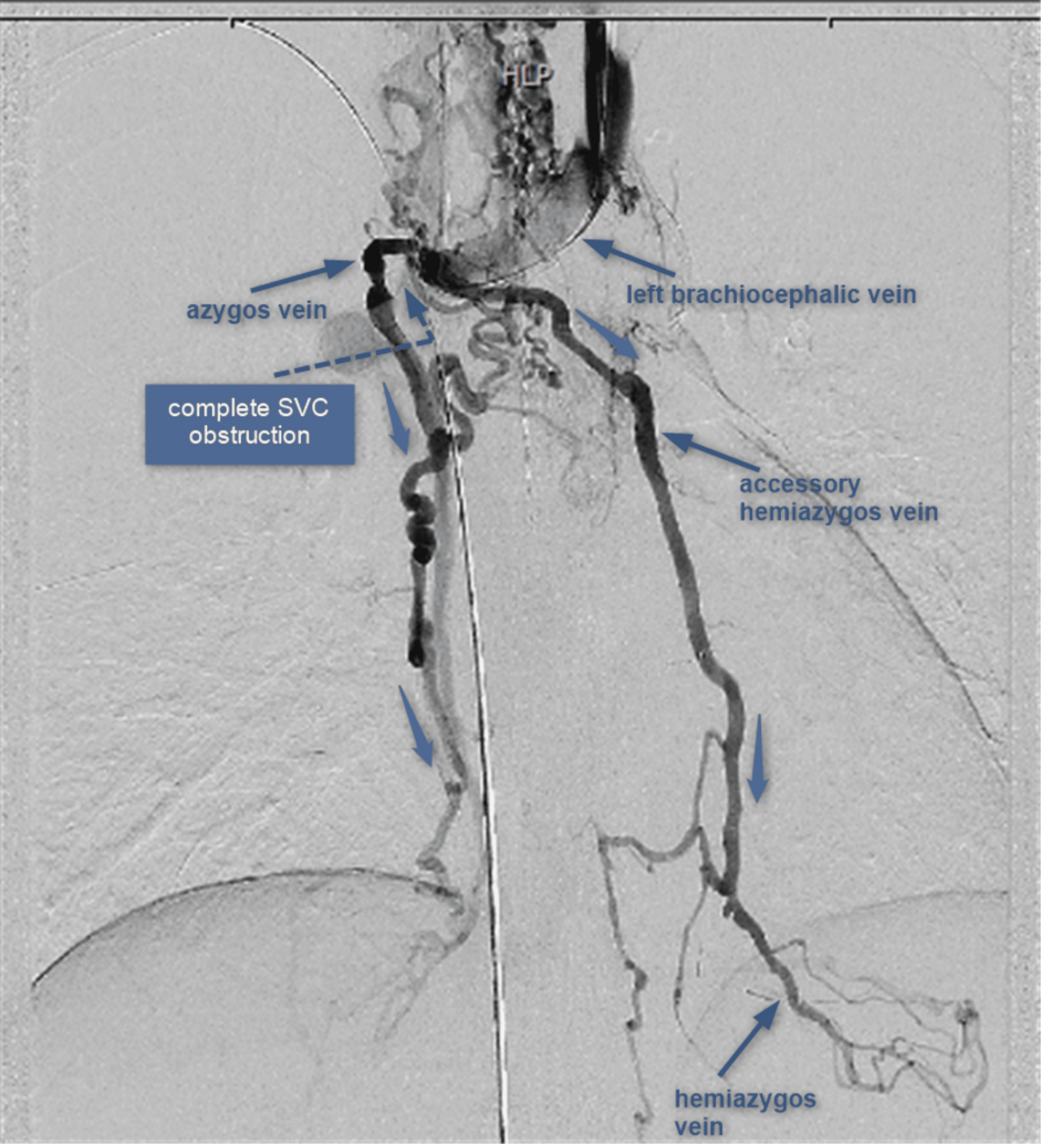
Digital subtraction venography showing complete occlusion of the superior vena cava (SVC) and brachiocephalic veins with poor filling of azygos and hemiazygos veins, and inferior vena cava. Esophageal veins are filling via accessory hemiazygos veins.

The diagnosis of downhill varices was made, and an angiography was performed (Figure 3).

**Figure 4:**
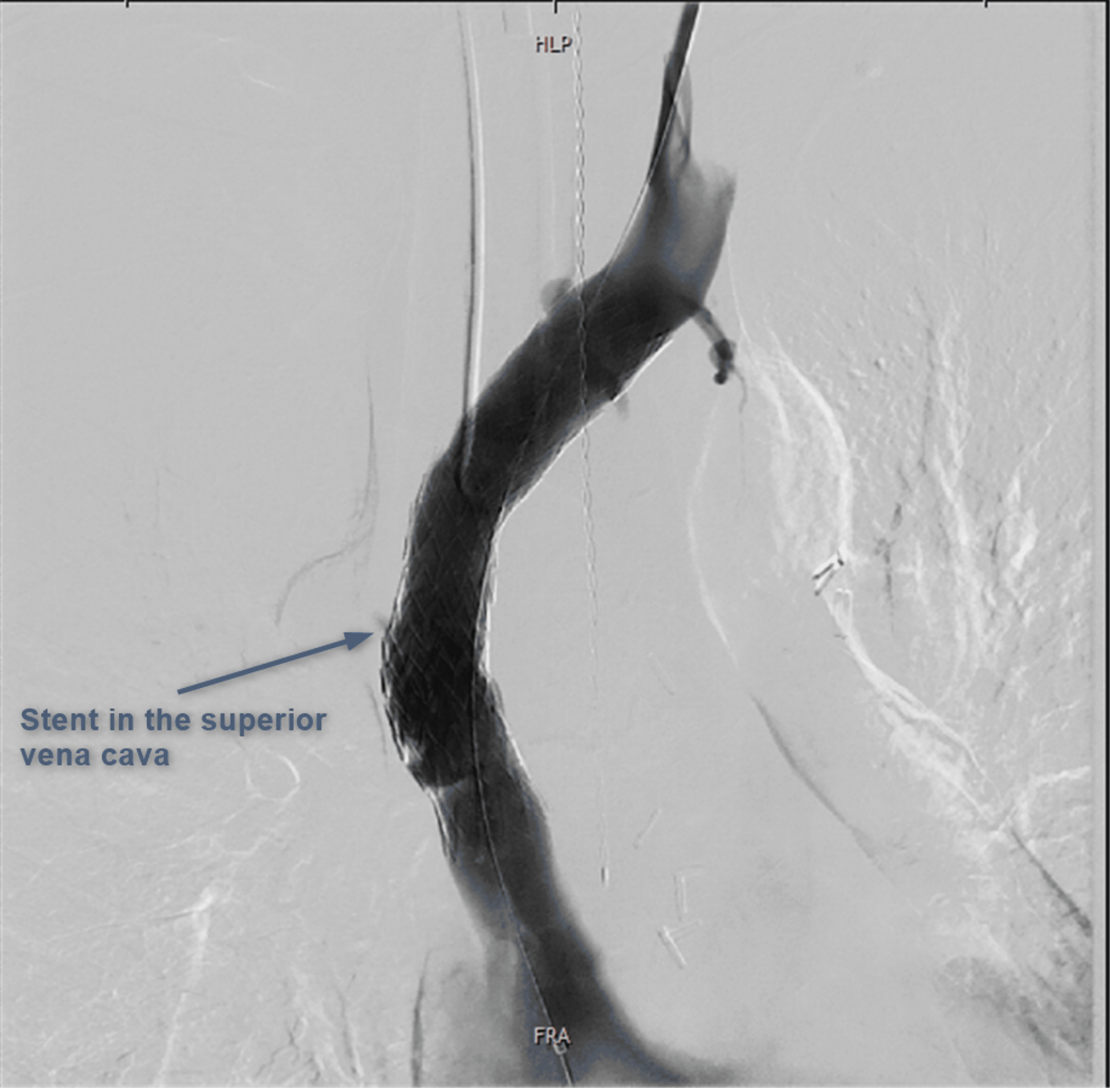
Venography demonstrating successful recanalization of the superior vena cava. No collateral flow into accessory hemiazygos veins is detectable after stent placement into superior vena cava and left brachiocephalic vein.

During a single endovascular procedure, 1) dialysis catheter removal, 2) stent recanalization of the left-sided brachiocephalic vein and SVC, and 3) placement of a new dialysis catheter were performed. First, the right-sided tunneled dialysis catheter was removed over the wire. The wire was then externalized from the inferior vena cava through right common femoral vein access using a snare catheter (pull-through maneuver). Second, percutaneous transluminal high-pressure balloon angioplasty and stent placement (BeYond Venous Self-Expanding Stent, 14/80 mm, Becton Dickinson, Franklin Lakes, NJ) were successfully performed from femoral access (Figure 4).

Finally, a new tunneled dialysis catheter was inserted through the freshly implanted stent to the beginning of the right atrium for ongoing dialysis access.

At 11-month follow-up, the patient remained clinically stable with no further bleeding episodes and no signs of facial edema. Duplex sonography confirmed a patent venous stent. Follow-up esophagogastroduodenoscopy showed no recurrence of esophageal varices.

## Discussion

Downhill esophageal varices are often overlooked due to their rarity, accounting for 0.4-10% of esophageal varices [3]. Bleeding from downhill varices is extremely rare, occurring in less than 0.1% of cases [4]. Only a few cases have been reported in the literature to date. Extensive venous obstruction of the SVC and brachiocephalic veins due to long-term indwelling, large-lumen dialysis catheters may limit the development of typical collateral veins into the inferior vena cava via the main azygos and hemiazygos veins. In our patient, small, atypical accessory hemiazygos veins were detected as feeders of esophageal veins (Figure 2).

Following stent placement, these collaterals were no longer visible at venography, and the patient had no more esophageal bleeding events during follow-up.

Traditional measures such as band ligation or sclerotherapy may achieve temporary hemostasis but rarely provide a durable solution. For this reason, endovascular recanalization with angioplasty and stent implantation is increasingly recognized as the gold standard for benign SVC obstruction. Published case series have demonstrated high technical success and low complication rates, with rapid symptom relief and sustainable venous drainage [5,6]. This case adds to the limited body of literature on downhill varices, demonstrating that addressing the venous obstruction can lead to complete resolution of variceal bleeding and therefore improve the quality of life of patients.

## Conclusions

Downhill esophageal varices are a rare and easily overlooked cause of upper gastrointestinal bleeding, particularly in patients without any signs of liver disease. In such cases, hematemesis or melena should prompt evaluation for possible obstruction of the SVC. When SVC obstruction is identified, management should focus on treating the underlying cause rather than relying solely on endoscopic measures. As demonstrated in this case, endovascular recanalization and stent placement can achieve definitive and sustained resolution of bleeding.

## References

[1] Cheng S (2009). Superior vena cava syndrome: a contemporary review of a historic disease. Cardiol Rev.

[2] Alimi YS, Gloviczki P, Vrtiska TJ ( 1998). Reconstruction of the superior vena cava: benefits of postoperative surveillance and secondary endovascular interventions. J Vasc Surg.

[3] Nguyen LP, Sriratanaviriyakul N, Sandrock C (2025). A rare but reversible cause of hematemesis: “downhill” esophageal varices. Case Rep Crit Care.

[4] Siegel Y, Schallert E, Kuker R (2015). Downhill esophageal varices: a prevalent complication of superior vena cava obstruction from benign and malignant causes. J Comput Assist Tomogr.

[5] Chakinala RC, Kumar A, Barsa JE (2018). Downhill esophageal varices: a therapeutic dilemma. Ann Transl Med.

[6] Karakhanian WK, Karakhanian WZ, Belczak SQ (2019). Superior vena cava syndrome: endovascular management. J Vasc Bras.

